# Substance Use and Mental Health during the First COVID-19 Lockdown in Germany: Results of a Cross-Sectional Survey

**DOI:** 10.3390/ijerph191912801

**Published:** 2022-10-06

**Authors:** Daniel Deimel, Christine Firk, Heino Stöver, Nicolas Hees, Norbert Scherbaum, Simon Fleißner

**Affiliations:** 1 German Institute for Addiction and Prevention Research, Catholic University of Applied Sciences, 52066 Aachen, Germany; 2 LVR-Hospital Essen, Department of Addictive Behavior and Addiction Medicine, Medical Faculty, University of Duisburg-Essen, 47057 Essen, Germany; 3 Institute of Health Research and Social Psychiatry, Catholic University of Applied Sciences, 52066 Aachen, Germany; 4 Institute for Addiction Research, Frankfurt University of Applied Sciences, 60318 Frankfurt, Germany

**Keywords:** COVID-19, substance use during lockdown, mental health during lockdown

## Abstract

Background: The measures taken to contain the COVID-19 pandemic have led to significant changes in people’s daily lives. This paper examines changes in substance use during the first lockdown (March–July 2020) and investigates mental health burdens in substance users with increased consumption of alcohol, nicotine or tetrahydrocannabinol (THC) in Germany compared to users with unchanged or reduced consumption. Method: In a cross-sectional online survey, 2369 people were asked about their mental health and their substance use during the first lockdown in Germany. Results: Of the participants, 28.5% increased their alcohol use, 28.8% their use of tobacco products, and 20.6% their use of THC-containing products during the pandemic. The groups with increased alcohol, nicotine, and THC use during the first lockdown reported more depressive symptoms and anxiety. Individuals who reported increased consumption of alcohol or nicotine were also more likely to experience loneliness and have suicidal thoughts and were more often stressed due to social distancing. Conclusion: Alcohol, nicotine and THC increased in a subgroup of consumers who reported to have more mental health problems compared to individuals who did not increase their consumption. This increased substance use could, therefore, be understood as a dysfunctional strategy to cope with negative emotions during the lockdown.

## 1. Introduction

The global disease (COVID-19) pandemic was and is a challenge for individuals’ daily lives. To date, over 267 million people have been infected with the virus and over 5.2 million people have died from or with the disease [1].

In Germany, the virus was confirmed for the first time on 27 January 2020 [2]. On 16 March 2020, measures to contain the coronavirus were adopted by the German government. The measures to restrict contact became effective on 22 March 2020 [3]. Contact was only allowed with persons from one’s own household. Private contact with another person outside one’s household was permitted only in public spaces. Private and public parties were prohibited and violations were sanctioned. Restaurants and bars were closed except for pick-up and delivery services. Likewise, all personal care facilities had to be closed. Effective hygiene concepts were mandatory in businesses, especially those open to the public [3]. The consequences of these measures were that social contacts were limited to an absolute minimum. These comprehensive restrictions were an attempt to prevent the spread of the virus. The measures had a profound effect on the everyday life of the population.

Several studies found an impact of the COVID-19 pandemic and its myriad disruptions on mental health [4,5,6,7,8]. Risk factors for increased psychological distress such as female gender, negative affect, social disconnectedness, and poor self-rated health status were identified in various studies [9,10,11,12,13]. A systematic review finds that at least the short-term effects of the COVID-19 pandemic are associated with a general worsening of mental health regardless of country or gender [14].

Previous studies reported an association between mental health problems and use of substances such as alcohol, nicotine or other drugs [15]. Further an increase in mental distress increases substance use, particularly following disasters [16]. For changes in substance use during the COVID-19 pandemic, no clear European trend seems to emerge from the report of the European Monitoring Centre for Drugs and Drug Addiction (EMCDDA) [17]. For Great Britain, it is reported that excessive alcohol consumption has become more frequent [18]. In Belgium, slightly increased consumption of alcohol and cigarettes is reported, but no change in the use of tetrahydrocannabinol (THC) [19]. In Germany, the consumption of alcohol seems to have increased somewhat during the first lockdown [20]. Another study suggests that in particular binge drinking increased in Germany [21]. Women seem to be particularly affected by increased consumption of alcohol and nicotine [22]. A recent review reports a trend towards increased consumption of alcohol and other substances during the pandemic, which were negatively correlated with mental health [23].

One explanation for the increase in substance use during the COVID-19 pandemic might be that substances are used as a coping strategy to regulate mental distress associated with the pandemic [24,25,26,27]. However, the impact of the pandemic on substance use and mental health on nicotine, alcohol, and THC users is still not fully comprehended. Therefore, the main aim of the current study is to investigate substance use in the general population and to compare the mental health of individuals with an increase in substance use since the pandemic with individuals with unchanged or decreased substance use. For taking effective measures, health care providers (e.g., counsellors, psychotherapists, physicians) need more detailed information on the impact of the lockdown on mental health in substance users. Based on previous findings, we hypothesize that individuals with an increase in substance use will also report an increase in depression, anxiety, suicidal ideation, loneliness, and a higher burden of social distancing.

## 2. Material and Methods

### 2.1. Design and Sampling

A cross-sectional online survey collected data on drug use and mental health during the first lockdown in Germany. The data were collected from 1 June 2020 until 17 July 2020. The questionnaire format was designed using the public survey tool LimeSurvey (https://www.limesurvey.org, accessed on 3 October 2022). The survey was conducted online and advertised through many websites (German AIDS Service Organizations, German Society for Social Psychiatry, German Federation of Telephone Emergency Services, German Federation for Social Work in the Healthcare System, German Society for Social Work in Addiction Aid). Anyone who was at least 18 was invited to participate. The survey was voluntary and anonymous, and participants could withdraw from the study at any time. No compensation was paid for participation. The questionnaire was available only in German language, therefore sufficient knowledge of the German language was needed. In total, 3154 people were reached through the online survey. For this evaluation, the subset of people who gave at least their age were included (*n* = 2369). Given that participants were able to stop and save their data at any point of the survey and the survey software was not programmed in a “forced choice” format, data of non-completers were included on a pairwise basis, resulting in a different number of responses per analysis. Potential bias will be discussed in Section 5, “Limitations”.

### 2.2. Measures

The survey started with a short introduction to the questionnaire. Subsequently, socio-demographic questions, questions on substance use and questions on mental health were asked with 131 items. Substance use was surveyed by a 12-month prevalence and with questions on changes in substance use during the lockdown, for alcohol, nicotine and illegal drugs, in particular THC. To measure changes in substance use, participants reported whether their consumption decreased, increased or did not change during the lockdown. In addition, it was asked whether it has become easier or more difficult to buy illicit drugs. Mental health was surveyed with the German version of the Brief Patient Health Questionnaire (PHQ-D) [28]. We used the subscale Patient Health Questionnaire (PHQ-9) [29] to measure depressive symptoms and the Generalized Anxiety Disorder Scale-7 (GAD-7) [30] to assess anxiety disorders. The PHQ-9 scale assesses the severity of depressive symptoms with a maximum score of 27 points. GAD-7 measures symptoms of anxiety with a maximum score of 21 points. A score of 10 points or above on each of the two scales indicates at least clinical significant depressive symptoms and anxiety. The internal reliability of the PHQ-9 was with a Cronbach’s α of 0.90 excellent. Additionally, the internal reliability of the GAD-7 was excellent (Cronbach’s α = 0.91). With the 11-item De Jong Giervald Loneliness Scale we surveyed emotional and social loneliness [31]. The scale assesses loneliness on a scale of 0–11 points. The cut-off score of 3 points and above indicates significant symptoms of loneliness [31]. The internal reliability of the De Jong Giervand Loneliness Scale was with a Cronbach’s α = 0.81 good.

In addition, specific effects of the lockdown on mental health were explored. For this purpose, the subjective burden of social isolation during lockdown was surveyed by a Likert scale. We asked the question: “How much do you feel socially isolated by the social distancing measures?” (0 = not isolated at all, 6 = very isolated). The frequency of suicidal thoughts during the lockdown were asked as follows: “From the time of social distancing/social restrictions due to the Corona pandemic, how often did you think about killing yourself?” (never, rarely (once), sometimes (twice), often (3–4 times), very often (5 times or more)).

### 2.3. Statistical Analysis

For data analyses, IBM SPSS Statistics version 25 (https://www.ibm.com/de-de/products/spss-statistics, accessed on 3 October 2022) was used. To compare groups with increased vs. unchanged substance use, independent *t*-tests were used for normally distributed data. For non-normally distributed or ordinal scaled data, Mann–Whitney-U-tests were used. Chi-square tests were used to compare the distributions of categorical variables. Significance was tested at the 5% level.

## 3. Results

### 3.1. Sociodemographics

The analysis of the survey included 2369 persons (female (67.8%), male (30.9%), and diverse (1.5%)) who provided at least information on age. The respondents were fairly evenly distributed across the age groups from 25 to 64. The sample was highly educated with 51.8% holding a university degree and 35.2% having completed secondary education. Furthermore, 61.5% were employed full-time or part-time. The majority of respondents had no children (73.7%). For all sociodemographic data see Table 1.

### 3.2. Mental Health

Depressive symptoms above the cut-off (>10 points) were reported by 25.9% of the sample. Generalized anxiety symptoms above the cut-off for at least moderate anxiety symptoms (>10 points) were reported by 23.3%. Loneliness above the cut-off (>3 points) were reported by 23.3%. At least having suicidal thoughts once during the lockdown were reported by 38.2%. For all mental health data see Table 2.

### 3.3. Drug Consumption during the First Lockdown

The respondents provided information on their alcohol, nicotine and/or THC consumption during the first lockdown. Table 3 gives the sociodemographic data of persons who had consumed any of the respective substances at least once in the last 12 months. In Table 4, the consumption data have been grouped into three categories (no consumption/less consumption, constant consumption, and more consumption). During the lockdown, 28.5% reported increased alcohol consumption, 28.8.% an increased nicotine consumption, and 20.6% reported an increased THC consumption.

In addition, 15% of the respondents reported that it was more difficult to purchase THC. Data on drug purchases for THC and other illicit drugs through the course of the lockdown is presented in Table 5.

### 3.4. Alcohol and Mental Health

Those respondents reporting increased alcohol consumption reported more depressive symptoms (PHQ-9) (*t*(1044) = −6.891, *p* < 0.001) compared with the group with similar or less self-reported alcohol consumption. The same was observed with regard to symptoms of anxiety disorder (GAD-7) (*t*(1059) = −7.584, *p* < 0.001) and with regard to loneliness (*t*(1123) = −4.869, *p* = 0.001). The group with increased alcohol consumption also reported more significant financial losses under the COVID-19 pandemic (X^2^ = 9.162, *p* < 0.002, df = 1), more burdens of social distancing (*U* = 405,484.500, *Z* = −6.132, *p* < 0.001) and more suicidal thoughts during lockdown (*U* = 70,896.500, *Z* = −2.904, *p* < 0.004). For detailed data, see Table 6.

### 3.5. Nicotine and Mental Health

The group with more nicotine consumption reported more depressive symptoms (PHQ-9) than the group with similar or less nicotine consumption (*t*(677) = −5.816, *p* < 0.001). This was also found with regard to the anxiety (*t*(668) = −7.013, *p* < 0.001) and loneliness during the lockdown (*t*(719) = −4.146, *p* < 0.001). The group with increased nicotine consumption also reported more burdens of social distancing (*U* = 66,379.500, *Z* = −5.091, *p* < 0.001) and more suicidal thoughts during lockdown (*U* = 17,111.000, *Z* = −2.203, *p* < 0.001). For detailed data, see Table 7.

### 3.6. THC and Mental Health

The group with more THC consumption showed more symptoms of depression (PHQ-9) compared to the group with unchanged or decreased THC consumption (*t*(162.079) = −2.568, *p* < 0.006). Similarly, those with less or unchanged THC consumption reported less anxiety measured with the GAD-7 (*t*(265) = −2.178, *p* < 0.015). For detailed data, see Table 8.

## 4. Discussion

The first aim of the current study was to investigate the prevalence rates of substance use (i.e., alcohol, nicotine and THC) during the pandemic in the German population.

In the current study, 28.5% reported drinking more alcohol than before the lockdown. At the same time, however, 30.3% reported consuming less alcohol during the lockdown than before. Therefore, it cannot generally be stated that alcohol consumption increased during the lockdown for most consumers as the consumption pattern seems to be more complex. These results are also in line with previous findings by Manthey and colleagues showing on average lower alcohol consumption during the first lockdown in Germany, but an increase in alcohol use in vulnerable groups [21]. A study from Ireland found that 15% of their participants reported an increase in alcohol consumption whereas 66% reported a decrease [32]. Koopman and colleagues, on the other hand, reported increased alcohol consumption in the general population in Germany [33], which was also found for consumers in the United States [34]. The current and previous findings, thus, point to an increase in alcohol use in at least a subgroup of users.

With regard to nicotine consumption, 28.8% of the respondents reported an increase in nicotine consumption whereas 32.9% reported a decrease in nicotine consumption. In line with our findings, a representative study on the prevalence of tobacco smoking in the adult population in Germany found an increase in smoking prevalence from 26.5% up to 30.9% [35]. In contrast, Damerow and colleagues reported less tobacco smoking during the lockdown [36]. Interestingly, Bommele et al. found that stress during the pandemic either predicted increased or decreased smoking behavior [37]. They postulate that the differential association between stress and smoking behavior during the pandemic might result from the fear of severe illness related to COVID-19, which, in turn, might have increased the motivation to stop smoking in some individuals whereas in others the burdens related to restrictions during the lockdown might have increased smoking behavior.

Regarding THC consumption, 52.9% decreased THC use and only 20.6% increased their consumption. This is in line with a study by Merrill et al. who found that THC use decreased in the subgroup of students during the lockdown [38]. They assume that this might be related to moving from campus to the parents’ home during the lockdown. In contrast, in a survey by Werse and Kamphausen exploring THC use in frequent users, they found that most of the respondents increased their THC consumption during the lockdown [39]. In contrast, Vanderbruggen et al. found that THC use did not change during the lockdown [19]. Thus, there is no clear picture related to the impact of the pandemic on THC consumption. Nevertheless, although most studies do not report a general increase in THC consumption but rather a decrease or no change in consumption, there seems to be a small subgroup with increased THC use during the lockdown.

The second aim of the current study was to investigate differences in mental health in individuals with increased substance use compared to individuals with unchanged or decreased substance use. As already noted, in the current study, substance use did not increase for all consumers. However, a subgroup of users reported increased use of alcohol, nicotine and THC, whereas others reported a decrease or no change in substance use. Given the high burden of disease associated with alcohol, nicotine and THC consumption [40,41,42,43,44], exploring factors that might be associated with an increase in substance use is highly warranted.

For alcohol and nicotine, individuals with an increase in substance use reported more depressive symptoms, anxiety and loneliness compared to individuals with less/unchanged substance use. Furthermore, they were more likely to report suicidal thoughts and experienced higher burdens due to social isolation. This is in line with findings from Australia showing an association between depression, anxiety and stress with increased alcohol and nicotine consumption during the lockdown [45]. In the current study, THC users with an increase in THC use reported more depressive symptoms and anxiety compared to users with unchanged consumption but no differences were found with respect to loneliness, suicidal thoughts or burdens related to social isolation between these groups.

The current findings are in line with previous findings pointing to an association between substance use and mental health problems or loneliness during the COVID-19 pandemic. Vanderbruggen and colleagues found that substance use correlates with feelings of loneliness and a loss of daily structure [19]. Faris and colleagues report that in Spain increased substance use is more likely among people with more depressive symptoms [25]. In a study from Greece, a significant proportion of respondents reported using alcohol and nicotine to cope better with anxiety and depression during the COVID 19 pandemic [26]. In a survey in France, Rolland and colleagues analyzed that lower well-being and increased stress are factors that increase behaviors related to addictions such as the use of alcohol and nicotine [27]. In an Australian study, 12.3% reported using drugs to cope with stress and emotions during the pandemic [24]. A cross-sectional study from UK also found increased alcohol consumptions predicted poor mental health [46].

Thus, the current and previous findings suggest that substance use may be used as a strategy to cope with mental health burdens during the pandemic. However, as we did not investigate whether there was a change in mental health compared to the time before the lockdown and data were acquired cross-sectionally, more research and cautious interpretation in the meantime is certainly needed.

## 5. Limitations

Some limitations warrant cautious interpretation of the data. The current sample is not representative for the general population in Germany due to the online recruiting process via different websites. The demographic data clearly show that both women (67.3%) and persons without children in the household (73.7%) are significantly overrepresented. Especially the factor of loneliness can thus lead to a distorted picture. Furthermore, women might be more affected by the impact of the pandemic than men [22]. In addition, our sample is highly educated. In addition, elderly people are underrepresented, which could be a result of the online recruiting process.

It should also be noted that this study did not ask if there was any change in mental health compared to the time before the lockdown, which was found in previous studies during the lockdown [47,48]. With regard to substance use, the survey provides insight into the change in substance use but gives no information about the absolute amount consumed. Further, the retrospective design of this cross-sectional study might be prone to recall biases.

## 6. Conclusions

To sum up, alcohol, nicotine and THC use did not increase in most of the users during the lockdown. However, there are subgroups with increased substance use. We assume that people suffering from greater psychological burdens during the lockdown are more vulnerable to increase their consumption. This increased substance use could, therefore, be understood as a strategy to cope with negative emotions during the lockdown, which could lead to additional problems. It is therefore necessary to identify vulnerable groups in times of lockdowns to promote mental health, which might also positively affect substance use. Prevention strategies thus need to address specific health needs and coping mechanisms. Additionally, methods of approaching these groups should be reflected; outreach work and target-group-specific social media campaigns could help to diminish social isolation. It is conceivable both to alleviate the psychological stress and to offer and enable other ways of coping with stress through more tailored services. The Robert Koch Institute (RKI) suggests that long-term consequences for mental health follow from prolonged stress, especially if stressors are not adequately coped with [49]. This paper shows that despite small changes in substance use in the general population, the consumption of alcohol, nicotine and THC needs to be addressed to ensure that pandemic-related burdens do not have long-term negative effects on mental health and substance (ab)use. Further research is needed to identify vulnerable groups and to develop and implement appropriate interventions.

## Figures and Tables

**Table 1:** Sociodemographic data of the entire sample.

	*n*	%
**Gender Identity**	2327	
Female	1595	67.3
Male	732	30.9
Diverse	35	1.5
**Age**	2369	
18–24	351	14.8
25–34	485	20.5
35–44	477	20.1
45–54	435	18.4
55–64	439	18.5
≥65	182	7.7
**Education**	2359	
University or university of applied sciences diploma	1228	51.8
Completed vocational education	285	12.0
Completion of secondary school	833	35.2
Other/none	13	0.5
**Employment status**	2363	
Full-time employed	853	36.0
Part-time employed	605	25.5
Student	418	17.6
Retired	247	10.4
Unemployed	94	4.0
Other	146	6.2
**Monthly net income**	2284	
<1000 Euros	604	25.5
1000–2000 Euros	742	31.3
2000–3000 Euros	599	25.3
More than 3000 Euros	339	14.3
**Financial losses due to the Corona pandemic**	2360	
no financial losses	1600	67.5
very small financial losses	401	16.9
significant financial losses	243	10.3
very strong financial losses	62	2.6
the income has completely disappeared	54	2.3
**Children in household**	2354	
0	1746	73.7
1	241	10.2
2	269	11.4
3	74	3.1
≥4	24	0.9
**Preexisting chronic conditions**		
cardiovascular diseases/hypertension	335	14.1
weakened immune system	261	11.0
high obesity	241	10.2
chronic respiratory diseases	173	7.3
diabetes mellitus	88	3.7
cancer	74	3.1
chronic liver diseases	21	0.9

**Table 2:** Mental health.

	M	MD	SD	Min	Max	>Cut-Off
Depressive Symptoms (PHQ-9)	7.1	6	5.9	0	27	25.9%
Generalized Anxiety (GAD-7)	6.6	5	5.3	0	21	23.3%
Loneliness	1.6	1	2.2	0	11	23.3%
Burdens of social isolation during lockdown	3.5	3	1.4	1	6	--
	*n*			%		
Suicidal thoughts during lockdown	938					
Never	580			61.8		
rarely (once)	193			20.6		
sometimes (2 times)	92			9.8		
often (3–4 times)	31			3.3		
very often (5 times or more)	42			4.5		

**Table 3:** Sociodemographic data of persons who used alcohol, nicotine and THC in the last 12 months.

	Alcohol Consumption Last 12 Months *n* = 2207		Nicotine Consumption Last 12 Months *n* = 920		THC Consumption Last 12 Months *n* = 478	
	*n*	%	*n*	%	*n*	%
**Gender Identity**	2148		895		465	
Female	1458	66.1	565	63.1	267	57.4
Male	659	29.8	311	34.7	180	38.7
Diverse	31	1.4	19	2.1	18	3.9
**Age**	2144		896		464	
18–24	327	15.3	164	18.3	121	26.1
25–34	446	20.8	237	26.5	147	31.7
35–44	434	20.2	180	20.1	89	19.2
45–54	388	18.1	173	19.3	61	13.1
55–64	391	18.2	118	13.2	42	9.1
≥65	158	7.4	24	2.7	4	0.9
**Education**	2145		890		462	
University or university of applied sciences diploma	1145	53.4	405	45.5	212	45.9
Completed vocational education	249	11.6	116	13.0	55	11.9
Completion of secondary school	743	34.6	367	41.2	195	42.2
Other/none	8	0.4	2	0.2	0	0.0
**Employment status**	2202		918		478	
Full-time employed	809	36.7	338	36.8	170	35.6
Part-time employed	567	25.7	225	24.5	107	22.4
Student	401	18.2	207	22.5	131	27.4
Retired	215	9.8	46	5.0	16	3.3
Unemployed	79	3.6	49	5.3	22	4.6
Other	131	5.9	53	5.8	32	6.7
**Monthly net income**	2076		881		458	
<1000 Euros	541	26.1	261	29.6	165	36.0
1000–2000 Euros	673	32.4	310	35.2	153	33.4
2000–3000 Euros	549	26.4	208	23.6	100	21.8
More than 3000 Euros	313	15.1	102	11.6	40	8.7
**Financial losses due to the Corona pandemic**	2200		917		477	
no financial losses	1487	67.6	585	63.8	289	60.6
very small financial losses	381	17.3	164	17.9	80	16.8
significant financial losses	222	10.1	115	12.5	68	14.3
very strong financial losses	61	2.8	32	3.5	18	3.8
the income has completely disappeared	49	2.2	21	2.3	22	4.6
**Preexisting chronic conditions**	2207		920		478	
cardiovascular diseases/hypertension	300	13.6	108	11.7	41	8.6
weakened immune system	225	10.2	108	11.7	74	15.5
high obesity	214	9.7	85	9.2	37	7.7
chronic respiratory diseases	155	7.0	69	7.5	38	7.9
diabetes mellitus	79	3.6	31	3.4	9	1.9
cancer	69	3.1	19	2.1	4	0.8
chronic liver diseases	15	0.7	6	0.7	3	0.6

**Table 4:** Substance use during first lockdown.

Substance Use	*n*	%
**Alcohol**		
Alcohol use last 12 months	2207	94.0
Alcohol use during lockdown	2184	
no consumption/less than before	661	30.3
unchanged	900	41.2
more than before	623	28.5
**Nicotine**		
Nicotine use last 12 months	920	45.6
Nicotine use during lockdown	907	
no consumption/less than before	298	32.9
unchanged	348	38.4
more than before	261	28.8
**THC**		
THC use last 12 months	478	23.5
THC use during lockdown	467	
no consumption/less than before	247	52.9
unchanged	124	26.6
more than before	96	20.6

**Table 5:** Drug purchase during lockdown.

Drug Purchase during Lockdown	THC (*n* = 187)		Amphetamine (*n* = 28)		Methamphetamine (*n* = 8)		Cocaine (*n* = 29)		Ecstasy (*n* = 25)	
	*n*	%	*n*	%	*n*	%	*n*	%	*n*	%
Easier than before the lockdown	4	2.1	2	7.1	1	12.5	1	3.4	2	8.0
Same as before the lockdown	155	82.9	18	64.3	4	50.0	23	79.3	17	68.0
More difficult than before the lockdown, at a lower or the same price	19	10.2	6	21.4	2	25.0	3	10.3	4	16.0
More difficult than before the lockdown, at a higher price	9	4.8	2	7.1	1	12.5	2	6.9	2	8.0

**Table 6:** Alcohol use and mental health.

Variable	No Increase in Alcohol Use			Increase in Alcohol Use			Test-Statistic				
								95% CI			Effect size
	*N*	M (SD)		*N*	M (SD)		*t*-test	LL	UL	*p*-value	Cohens d
**Age**	1258	43.04 (15.88)		606	40.36 (13.27)		3.83	1.31	4.06	0.001	0.19
**Mental health**											
Depression (PHQ-9 score)	1210	6.30 (6.63)		561	8.35 (5.91)		−6.89	−2.63	−1.47	0.001	0.37
Anxiety (GAD-7 score)	1190	5.81 (5.04)		571	7.85 (5.39)		−7.58	−2.57	−1.51	0.001	0.39
Loneliness (score)	1290	2.56 (2.42)		623	3.18 (2.68)		−4.87	−0.87	−0.37	0.001	0.25
	*N*	%		*N*	%		X^2^			*p*-value	Phi
**Gender**	2126										
Female	1069	70.4		375	61.8		16.69			0.001	0.089
Male	433	28.5		218	35.9						
Diverse	17	1.1		14	2.3						
**Monthly net income**	2056										
<1.000 Euros	386	26.3		150	25.5		0.49			0.921	
1.000–2.000 Euros	480	32.7		188	32.0						
2.000–3.000 Euros	387	26.4		158	26.9						
More than 3.000 Euros	215	14.6		92	15.6						
**Mental health**											
Depression (PHQ-9 ≥ 10)	1298	28.2		535	36.1		11.09			0.001	0.08
Anxiety (GAD-7 ≥ 10)	1436	21.5		571	34.0		33.76			0.001	0.13
Loneliness (cut-off ≥ 3)	671	43.1		313	50.2		9.25			0.002	0.07
**Significant financial losses from the Corona pandemic**	1556	4.1		621	7.2		9.16			0.002	0.07
	*N*	Mdn (IQR)	M	*N*	Mdn (IQR)	M	Mann–Whitney-U			*p*-value	*r*
**Burdens of social distancing**	1558	3 (2)	3.36	623	4 (2)	3.76	405,484.50			0.001	0.13
**Suicidal thoughts during lockdown**	577	1 (1)	1.59	275	1 (1)	1.81	70,896.50			0.004	0.10

**Table 7:** Nicotine consumption and mental health.

Variable	No Increase in Nicotine Use			Increase in Nicotine Use			Test-Statistic				
								95% CI			Effect size
	*N*	M (SD)		*N*	M (SD)		*t*-test	LL	UL	*p*-value	Cohens d
**Age**	450	40.08 (13.92)		254	37.96 (12.66)		1.99	0.34	4.19	0.023	0.16
**Mental health**											
Depression (PHQ-9 score)	435	7.24 (6.15)		244	10.12 (6.28)		−5.82	−3.86	−1.91	0.001	−0.47
Anxiety (GAD-7 score)	428	6.39 (5.41)		242	9.49 (5.65)		−7.01	−3.97	−2.23	0.001	−0.56
Loneliness (score)	460	2.81 (2.53)		261	3.62 (2.50)		−4.15	−1.19	−0.43	0.001	−0.32
	*N*	%		*N*	%		X^2^			*p*-value	Phi
**Gender**	883										
Female	399	63.5		160	62.7		0.61			0.739	
Male	217	34.6		88	34.5						
Diverse	12	1.9		7	2.7						
**Monthly net income**	869										
<1.000 Euros	181	29.3		75	29.9		7.39			0.060	
1.000–2.000 Euros	222	35.9		88	35.1						
2.000–3.000 Euros	134	21.7		69	27.5						
More than 3.000 Euros	81	13.1		19	2.2						
**Mental health**											
Depression (PHQ-9 ≥ 10)	180	32.4		112	46.9		14.96			0.001	0.14
Anxiety (GAD-7 ≥ 10)	140	23.2		112	46.3		44.09			0.001	0.23
Loneliness (cut-off ≥ 3)	302	46.7		161	61.7		16.60			0.001	0.14
**Significant financial losses from the Corona pandemic**	32	5.0		21	8.0		3.17			0.075	
	*N*	Mdn (IQR)	M	*N*	Mdn (IQR)	M	Mann–Whitney-U			*p*-value	*r*
**Burdens of social distancing**	645	3 (3)	3.44	261	4 (2)	3.97	66,379.50			0.001	0.17
**Suicidal thoughts during lockdown**	275	1 (1)	1.67	141	1 (1)	1.92	17,111.00			0.028	0.11

**Table 8:** THC consumption and mental health.

Variable	No Increase in THC Use			Increase in THC Use			Test-Statistic				
								95% CI			Effect size
	*N*	M (SD)		*N*	M (SD)		*t*-test	LL	UL	*p*-value	Cohens d
**Age**	182	33.86 (12.49)		94	34.87 (12.70)		0.62	−2.16	4.13	0.537	
**Mental health**											
Depression (PHQ-9 score)	172	8.42 (5.95)		92	10.63 (7.00)		−2.57	−3.90	−0.51	0.006	−0.35
Anxiety (GAD-7 score)	179	7.69 (5.64)		89	9.31 (5.95)		−2.18	−3.09	−0.16	0.015	−0.28
Loneliness (score)	189	3.26 (2.45)		96	3.70 (2.43)		−1.42	−1.04	−0.17	0.079	
	*N*	%		*N*	%		X^2^			*p*-value	Phi
**Gender**	454										
Female	209	58.2		54	56.8		0.07			0.968	
Male	136	37.9		37	38.9						
Diverse	14	3.9		4	4.2						
**Monthly net income**	448										
<1.000 Euros	130	36.7		35	37.2		3.06			0.383	
1.000–2.000 Euros	112	31.6		37	39.4						
2.000–3.000 Euros	79	22.3		16	17.0						
More than 3.000 Euros	33	9.3		6	6.4						
**Mental health**											
Depression (PHQ-9 ≥ 10)	136	41.2		44	51.2		2.75			0.097	
Anxiety (GAD-7 ≥ 10)	114	32.7		36	40.4		1.91			0.167	
Loneliness (cut-off ≥ 3)	207	55.8		61	63.5		1.87			0.171	
**Significant financial losses from the Corona pandemic**	28	7.6		12	12.5		2.36			0.124	
	*N*	Mdn (IQR)	M	*N*	Mdn (IQR)	M	Mann–Whitney-U			*p*-value	*r*
**Burdens of social distancing**	370	4 (2)	3.62	96	4 (2)	3.93	15,520.00			0.051	
**Suicidal thoughts during lockdown**	201	1 (1)	1.74	57	1 (2)	1.95	5264.50			0.295	

## Data Availability

The datasets generated and/or analyzed during the current study are not publicly available due to reasons of sensitivity but are available from the corresponding author Daniel Deimel on reasonable request.
